# Design and Synthesis of Non-Covalent Imidazo[1,2-*a*]quinoxaline-Based Inhibitors of EGFR and Their Anti-Cancer Assessment

**DOI:** 10.3390/molecules26051490

**Published:** 2021-03-09

**Authors:** Manvendra Kumar, Gaurav Joshi, Sahil Arora, Tashvinder Singh, Sajal Biswas, Nisha Sharma, Zahid Rafiq Bhat, Kulbhushan Tikoo, Sandeep Singh, Raj Kumar

**Affiliations:** 1 Laboratory for Drug Design and Synthesis, Department of Pharmaceutical Sciences and Natural Products, School of Health Sciences, Central University of Punjab, Bathinda 151401, Punjab, India; pharma.manvendra003@gmail.com (M.K.); garvjoshi@gehu.ac.in (G.J.); deepsahil7999@gmail.com (S.A.); sajal.143rx@gmail.com (S.B.); 2 School of Pharmacy, Graphic Era Hill University, Dehradun 248171, Uttarakhand, India; 3 Department of Human Genetics and Molecular Medicine, Central University of Punjab, Bathinda 151401, Punjab, India; sidhu.tash@gmail.com (T.S.); sandeepsingh82@gmail.com (S.S.); 4 Department of Pharmacology and Toxicology, National Institute of Pharmaceutical Education and Research, S.A.S. Nagar 160062, Punjab, India; nishasharma.nics8@gmail.com (N.S.); zbhat800@gmail.com (Z.R.B.); tikoo.k@gmail.com (K.T.)

**Keywords:** imidazo[1,2-*a*]quinoxaline, non-covalent EGFR inhibitors, anticancer agents, mutant EGFR inhibitors

## Abstract

A series of 30 non-covalent imidazo[1,2-*a*]quinoxaline-based inhibitors of epidermal growth factor receptor (EGFR) were designed and synthesized. EGFR inhibitory assessment (against wild type) data of compounds revealed **6b**, **7h**, **7j**, **9a** and **9c** as potent EGFR^WT^ inhibitors with IC_50_ values of 211.22, 222.21, 193.18, 223.32 and 221.53 nM, respectively, which were comparable to erlotinib (221.03 nM), a positive control. Furthermore, compounds exhibited excellent antiproliferative activity when tested against cancer cell lines harboring EGFR^WT^; A549, a non-small cell lung cancer (NSCLC), HCT-116 (colon), MDA-MB-231 (breast) and gefitinib-resistant NSCLC cell line H1975 harboring EGFR^L858R/T790M^. In particular, compound **6b** demonstrated significant inhibitory potential against gefitinib-resistant H1975 cells (IC_50_ = 3.65 μM) as compared to gefitinib (IC_50_ > 20 μM). Moreover, molecular docking disclosed the binding mode of the **6b** to the domain of EGFR (wild type and mutant type), indicating the basis of inhibition. Furthermore, its effects on redox modulation, mitochondrial membrane potential, cell cycle analysis and cell death mode in A549 lung cancer cells were also reported.

## 1. Introduction

**Figure 1:**
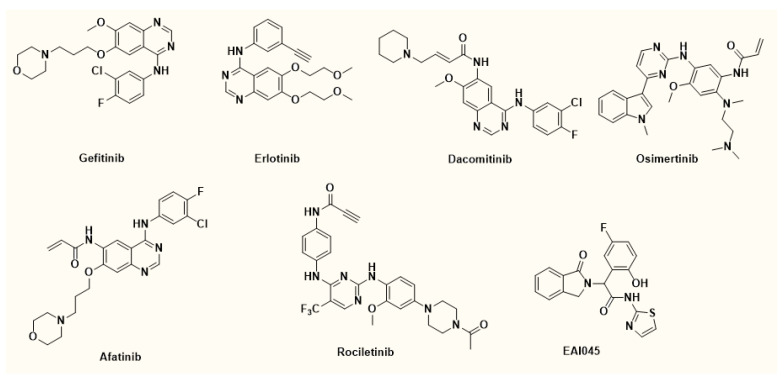
Chemical structures of FDA-approved epidermal growth factor receptor (EGFR) inhibitors.

Epidermal growth factor receptor (EGFR) protein is a well-studied oncological drug target as it is overexpressed in almost all types of cancers, including non-small cell lung cancer (NSCLC), breast, colon, prostate, etc. [1,2]. Quinazoline-based erlotinib and gefitinib were the first-generation EGFR inhibitors designed to competitively inhibit EGFR kinase activity (Figure 1) [3,4]. Their use provides an effective treatment to the NSCLC patients protecting the L858R activating mutation; however, their prolonged use imparts T790M mutation (gatekeeper residue mutation) in the kinase domain of EGFR, leading to the development of resistance in 60% of patients [4]. This led to the discovery of second-generation EGFR inhibitors (afatinib, dacomitinib), which are irreversible and effective in cancer with T790M mutation; however, they are associated with side effects. Consequently, third-generation EGFR inhibitors (rociletinib, osimertinib) were developed, which irreversibly target L858R/T790M mutated EGFR by making a covalent bond with Cys and sparing the wild type [5]. Unfortunately, the prolonged use of third-generation EGFR inhibitors led to drug resistance due to C797S mutation and toxicity [6,7]. Recently, fourth-generation EGFR inhibitor, an allosteric inhibitor EAI045 has been introduced, but its use is limited to the L858R-activating mutation [8] and is under clinical trial. Therefore, the development of novel non-covalent and reversible EGFR inhibitors with alternate scaffolds which do not involve covalent reaction with Cys797 is warranted [9,10,11].

**Figure 2:**
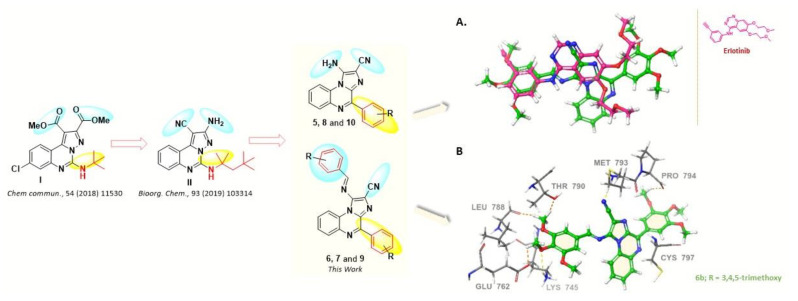
Design of target compounds (**5**–**10**): (**A**) Docked 3D-poses and overlay of designed compound (e.g., **6b;** R = 3,4,5-trimethoxy- a representative compound) and erlotinib showing similar binding modes at ATP binding domain of EGFR^WT^ (PDB: 3VJO); (**B**) 3D-interaction diagram of the designed compound with ATP binding pocket of EGFR^WT^ (PDB: 3VJO).

In the recent past, we were interested in developing the synthetic approaches and assessing the biological activities of pyrazolo[1,5-*c*]quinazolines [12,13,14,15], which eventually emerged as active EGFR inhibitors [16,17]. It was observed that polar substitutions (–CO_2_Me, –COAr, –CN and –NH_2_) on pyrazole (e.g., **I** and **II** of our previous studies) were critical for binding with EGFR residual amino acids [16,17]. To search for better EGFR inhibitors, we considered the ATP binding pocket of EGFR and pyrazolo[1,5-*c*]quinazoline pharmacophore and designed the new series of compounds (**5**–**10**) by (a) replacing the pyrazolo[1,5-*c*]quinazolines (**I** and **II**) with imidazo[1,2-*a*]quinoxaline template, (b) switching over the positions of –CN and –NH_2_ of **II** and/or making its imine formation and (c) removing metabolically soft spot, e.g., replacing *N*-alkyl of **I** and **II** with an aryl moiety. Furthermore, preliminary molecular docking studies of designed series (e.g., **6b** a representative compound; Figure 2) at EGFR–ATP pocket (EGFR^WT^ PDB ID: 3VJO) revealed that representative compound occupied the ATP binding pocket similar to erlotinib (Figure 2A); the –CN was found to make a hydrogen bond with a hydrogen of Met793, which is important for the inhibition of EGFR. The 3,4,5-trimethoxybenzylidene moiety of **6b** was oriented towards Thr790 (gatekeeper residue), Leu788, Glu762 and Lys745 (responsible for interaction with AMNP phosphate) and interacted with them through weak hydrogen bonds. Another C-2 substituted 3,4,5-trimethoxyphenyl was aligned in close proximity of Pro794 (weak hydrogen bond) and Cys797 (Figure 2B). Overall, binding pattern and docking score of the designed compound was better than **I** and **II** (See Supplementary Materials, Figure S1, Table S1). Furthermore, imidazole-fused [11,18] quinoxaline [19], in particular, imidazo[1,2-*a*]quinoxalines have gained significant interest due to their broad-spectrum activities [20,21], including IκB kinase (IKK) [22] and lymphocyte-specific protein tyrosine kinase (LCK) [23] inhibitory activities.

## 2. Results and Discussion

### 2.1. Chemistry

**Scheme 1:**
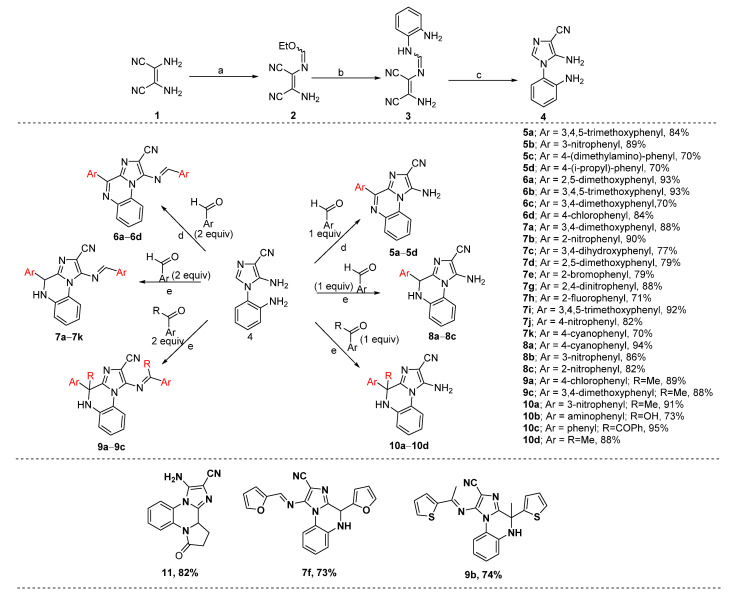
Synthesis of target compounds (**5**–**11**). Reagents and conditions: (**a**) CH(OEt)_3_, 1,4-dioxane, reflux, 6 h, 81%; (**b**) *o*-phenylenediamine, aniline hydrochloride, EtOH, rt, 5 h, 83%; (**c**) KOH (1M), H_2_O, rt, 89%; (**d**) *p*-TSA.H_2_O, MeOH, MW*/80 °C, 0.5 h; (**e**) *p*-TSA.H_2_O, MeOH, MW**/80 °C, 25 min. * Reaction was carried out at 200 W in an open condenser; ** Reaction was carried out at 200 W in a sealed tube.

As per Scheme 1, 30 target compounds (**5–11**) were prepared via microwave-assisted Pictet–Spengler (PS) reaction [24] of **4** with respective aryl aldehyde/ketone (1 or 2 equiv) in the presence of *p*-TSA (*p*-toluene sulphonic acid) as per our previous reports [25,26]. Intermediate **4** was synthesized via three step reactions which are as follows. 2,3-Diaminomaleonitrile (**1**) was heated with triethyl orthoformate in 1,4-dioxane to yield the compound **2**, which upon substitution reaction with the *o*-phenylenediamine afforded compound **3**. Compound **3** under basic condition underwent an internal cyclization reaction to produce intermediate **4.** All the synthesized target molecules were purified by column chromatography and characterized by NMR, HRMS, IR and melting point.

### 2.2. Biological Evaluation

#### In Vitro EGFR Kinase Inhibitory Activity and Antiproliferative Assay

All the target compounds were first assessed for their inhibitory potential of EGFR^WT^ by inhibiting ATP-dependent phosphorylation of EGFR. To our delight, five compounds **6b**, **7h**, **7j**, **9a** and **9c** inhibited EGFR^WT^ with IC_50_ values of 211.22, 222.21, 193.18, 223.32 and 221.53 nM, respectively, which were comparable to erlotinib (221.03 nM). However, compounds **5a**, **5c**, **5d**, **6c**, **6d**, **7a**–**e**, **7i**, **7k**, **8a**–**c**, **10b**–**d** and **11** were poorly active or possessed no activity at all (Table 1). 

**Figure 3:**
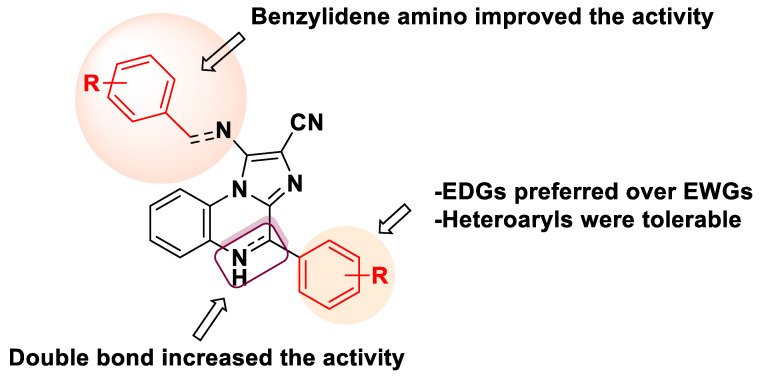
Illustration depicting the structure-activity relationship for the synthesized series (**5**–**11**).

From the EGFR inhibitory values, a limited structure–activity relationship was depicted as follows; a) in general, compounds having benzylidene amino functionality were potent inhibitors of EGFR (e.g., **6a**, **6b**, **7f–h**, **7j** and **9a**–**c**) except **5b** and **10a**. Furthermore, dihydroimidazo[1,2-*a*]quinoxalines were less potent as compared to their corresponding imidazo[1,2-*a*]quinoxalines (compare **8b**, **7i** and **7d** with **5b**, **6b** and **6a**, respectively) except for the pair **6c** and **7c**. Heteroaryl groups such as thieno and furano were found to be tolerable. Compounds with electron-donating groups possessed better activity than compounds with electron-withdrawing groups (e.g., **8a**–**c**). Furthermore, trimethoxy substitutions were preferred over dimethoxy groups (compare **6b** with **6a** and **6c**). Some of the observations are depicted in Figure 3.

Next, we analyzed the antiproliferative potential of most potent EGFR^WT^ inhibitors **6b**, **7h**, **7j**, **9a** and **9c** on heterozygous cancer cell lines harboring EGFR^WT^; A549 (lung), HCT-116 (colon) and MDA-MB-231 (breast) by performing MTT assay. A549 cells are major EGFR expressing cells derived from human lung carcinoma and possess adherent culture properties. HCT-116 cell lines are epithelial and are obtained from colon tissue of Homo sapiens, human, in the condition of colorectal carcinoma. This cell line is a suitable transfection host and has a mutation in codon 13 of the ras proto-oncogene, and can be used as a positive control for PCR assays of mutation in this codon (ATCC^®^). Furthermore, MDA-MB-231 cells are “basal” type and triple-negative (estrogen receptor, progesterone receptor and HER2 negative) breast adenocarcinoma cancer cell line that is aggressive. As expected, all EGFR^WT^ inhibitors possessed higher antiproliferative potential than the positive control (erlotinib) against at least two cancer cell lines (except **9a**, to which A549 cells were sensitive only and **9c**; Table 2) and were non-cytotoxic to normal cells (HBL-100 and HPBMCs) at the highest concentration of 10 µM incubated for 24 h (see Supplementary Materials, Figure S2).

**Figure 4:**
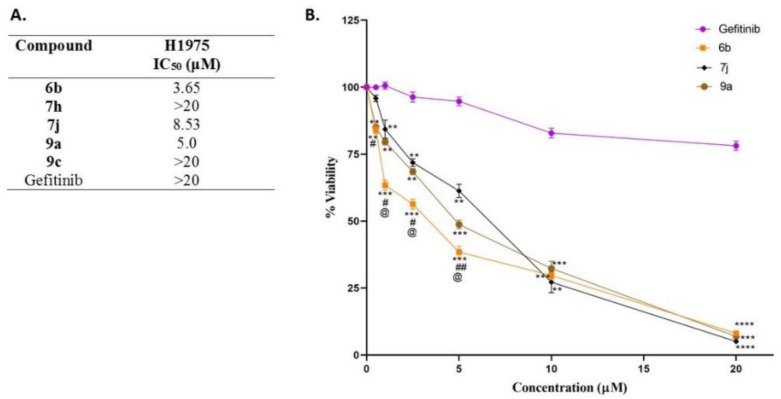
(**A**) IC_50_ values of EGFR inhibitors against non-small cell lung cancer (NSCLC) cell line H1975 carrying L858R + T790M double mutation (**B**). Dose–response curve for **6b**, **7j** and **9a** in comparison with gefitinib control. The percentage of viable cells is shown relative to that of untreated control cells. For statistical analysis of the data, GraphPad Prism 8.0.2 software (San Diego, CA, USA) was used. Data were presented as means ± standard error mean (S.E.M.) of three independent experiments. Two-way ANOVA was used for the comparison of multiple groups. Statistical significance was checked at * or # or @ *p* < 0.05, ** or ## *p* < 0.01, *** *p* < 0.001, **** *p* < 0.0001, where * represents difference vs. control group, # represents difference vs. compound **7j** and @ represents difference vs. **9a**.

All five compounds were subsequently investigated through antiproliferation assay employing a gefitinib-resistant NSCLC cell line H1975 harboring EGFR^L858R/T790M^. To our satisfaction, **6b**, **7j** and **9a** exhibited significant growth inhibitory activity with IC_50_ values of 3.65, 8.53 and 5.0 μM, respectively (Figure 4). Surprisingly, **7h** did not show comparable activity (>20 μM). Thus, overall results indicated that compounds were much more sensitive towards H1975 cells than gefitinib (>20 μM). Furthermore, **6b** may serve as a lead compound in the treatment of gefitinib-resistant EGFR mutant NSCLC.

### 2.3. Molecular Docking

**Figure 5:**
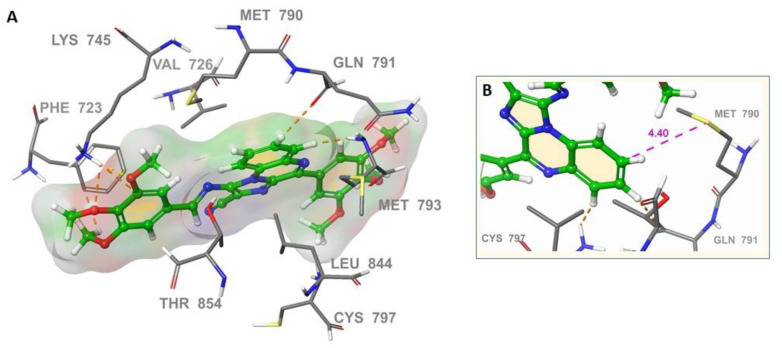
(**A**) 3D docking pose of **6b** at the active site of EGFR^L858R/T790M^ (PDB code 5C8K) showing binding and important interactions with some of the important amino acid residues. Some residues are removed for clarity; (**B**) close-in image of **6b** presenting the position of the imidazo[1,2-*a*]quinoxaline core and the methionine gatekeeper sulfur.

To understand the possible binding modes of compounds at the active site, flexible docking of the representative compound **6b** into the active site of EGFR^L858R/T790M^ (PDB: 5C8K; Figure 5A) was performed using Glide in Schrödinger. It was found that imidazo[1,2-*a*]quinoxaline core was aligned to mutant gatekeeper residue Met790 at a distance of 4.40 Å (Figure 5B). This was per the literature that aryl–methionine interactions are common and methionine sulfur is often situated in the same plane as the aryl ring [27,28]. Furthermore, the core was also observed to interact with the carbonyl oxygen of Gln791 via hydrogen bonding with sp^2^C–*H*. The ether oxygen atoms of 3-methoxy and 4-methoxyl of **6b** showed weak hydrogen bonding although within the range of hydrogen-bonding distance with the conserved catalytic Lys745 residue, at 1.8 Å and 2.39 Å distances (Figure 5A), respectively, whereas the same 3-methoxy was found to be at 3.97 Å distance (Figure 2B) in case of wild type EGFR, revealing more affinity of **6b** towards mutant type EGFR. The imidazole ring of **6b** was sandwiched in the pocket formed by Val726, Leu844 and Cys797.

### 2.4. Flow Cytometric Analysis of Reactive Oxygen Species (ROS), Mitochondrial Membrane Potential and Apoptosis

**Figure 6:**
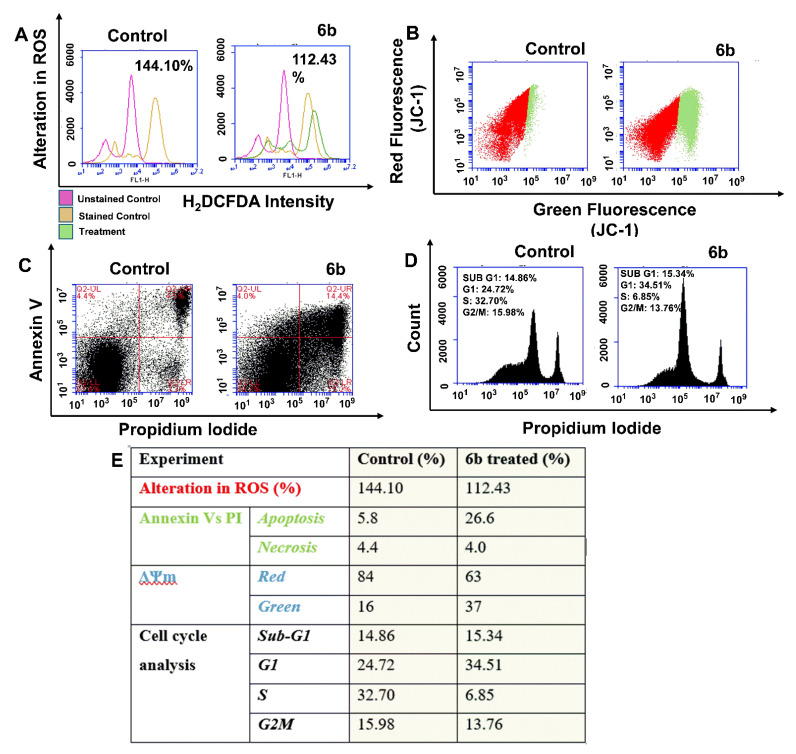
The assays were performed on A549 cells previously treated with investigational compound **6b** at sub-IC_50_ concentration for 48 h before analysis. Flow cytometry analysis (**A**) suggests alteration in ROS as indicated by H2DCFDA assay; (**B**) shows mitochondrial membrane depolarization (low red/green) as conferred by JC-1 assay; (**C**) depicts an apoptotic mode of cancer cell death as indicated by Annexin V vs. PI assay and (**D**) elaborates cell cycle arrest at G1 phase as indicated by PI assay. (**E**) Table summarizes the quantification values for experiments (**A**–**D**).

Considering the better EGFR inhibition and antiproliferative activity against mutant cells, we selected compound **6b** to understand the secondary anticancer mechanism elicited to induce cancer cell death at sub-IC_50_ in A549 cells. The results of flow cytometric analysis are collected in Figure 6 and summarized as follows. **6b** was found to reduce the oxidative stress (112.43%) inside the cancer cell as indicated by H_2_DCFDA assay (Figure 5A). A similar trend was observed in MCF-7 (breast) and MDA-MB-231 cells (Supplementary Materials, Figure S3). However, the percentage decrease in ROS was marginal in these two cell lines; the result still suggests that ROS level was kept under check by **6b** and was not elevated. This is important as most kinase inhibitors are reported to induce cardiotoxicity by upsurging the ROS level [29,30]. Furthermore, **6b** altered the mitochondria permeability by decreasing the membrane potential y (JC-1 assay; Figure 6B) and led to cancer cell death via apoptosis (PI vs. Annexin V assay; Figure 6C). **6b** induced cell cycle arrest at G1 phase (34.51%) by inhibiting the S phase (Figure 6D).

## 3. Experimental

### 3.1. Synthesis of Intermediates (***2–4***)

#### 3.1.1. *N*-(2-Amino-1, 2-dicyanovinyl)-formimydic acid ester (**2**) 

A mixture of 2,3-diaminomaleonitrile (3.0 g, 27.7 mmol, 1 equiv) and triethyl orthoformate (4.0 g, 27.0 mmol, 0.98 equiv) in 1,4-dioxane (25 mL) was heated for 6 h (TLC). The mixture was concentrated under reduced pressure, extracted with diethyl ether (40 mL × 3) and filtered. Yellow needle-shaped solid crystals of *N*-(2-amino-1, 2-dicyano-vinyl)-formimydic acid ester (**2**) were obtained after filtrate concentration. Yield: 78%; Color: Yellow; m.p.: 134–136 °C (135 °C Lit. [25]) **2** was used for the next step without further purification.

#### 3.1.2. *N*-(2-Amino-1,2-dicyanovinyl)-*N*’-(2-amino-phenyl)formimidine (**3**) 

A mixture of **2** (3.0 g, 18.28 mmol, 1 equiv) and *o-*phenylenediamine (1.58 g, 14.63 mmol, 0.8 equiv) in EtOH (4 mL) was stirred for 12 h (TLC) at rt. Brown colored solid *N*-(2-amino-1,2-dicyano-vinyl)-*N*’-(2-amino-phenyl)formimidine (**3**) was obtained after concentrating the reaction mixture. Yield: 81%; Color: Brown; m.p.: 119–121 °C (118–120 °C Lit. [25]) **3** was used for the next step without further purification.

#### 3.1.3. 5-Amino-1-(2-amino-phenyl)-1*H*-imidazole-4-carbonitrile (**4**) 

A suspension of **3** (3.0 g, 18.28 mmol, 1 equiv) and 1 M aq. KOH (15 mL) in H_2_O (1 mL) was stirred for 12 h (TLC) at rt. The mixture was extracted with EtOAc (20 mL × 3). The organic layer was washed with brine (20 mL) and dried over anhydrous sodium sulphate and concentrated under reduced pressure to afford crude 5-amino-1-(2-amino- phenyl)-1*H*-imidazole-4-carbonitrile (**4**) which was purified by recrystallization from EtOAc. Yield: 89%; Color: Yellowish solid; m.p.: 197–199 °C (196–198 °C Lit. [25]). The physical data of **2**–**4** was found to be in accordance with the literature [25].

### 3.2. Synthesis of Target Compounds (***5**–**10***)

#### 3.2.1. Representative Procedure for the Synthesis of (*E*)-1-((3,4,5-Trimethoxybenzylidene)amino)-4-(3,4,5-trimethoxyphenyl)imidazo[1,2-*a*]quinoxaline-2-carbonitrile (**6b**) 

In a reaction vial, to a suspension of **4** (100 mg, 0.502 mmol) in MeOH (1 mL) was added 3,4,5-trimthoxybenzaldehyde (199.9 mg, 1.004 mmol) and *p*TSA (1 mol %). The mixture was heated at 200 W in an open condenser under mw irradiation (Discover System; CEM, Matthews, NC, USA) at 80 °C for 0.5 h (TLC). MeOH was evaporated from mixture, concentrated using rotary evaporator, extracted with EtOAc (10 mL × 3), dried and purified by column chromatography to afford **6b.** Yield: 95%; Color: yellow; m.p.: 143–145 °C (142–144 °C Lit. [25]). IR (KBr, cm^−1^): 2227 (CN stretch), 1575 (C=N stretch), 1501 (C=C stretch) and 1127 (C–O stretch). ^1^H-NMR (400 MHz, CDCl_3_, TMS = 0) δ (ppm): 8.97 (2H, s), 8.12–8.09 (1H, m), 8.03 (2H, s), 7.64–7.55 (2H, m), 7.28 (2H, d, *J* = 8 Hz), 4.00 (15H, s), 3.93 (3H, s). ^13^C-NMR (100 MHz, CDCl_3_, TMS = 0) δ (ppm): 164.11, 153.74, 152.93, 149.02, 144.79, 143.14, 140.71, 136.87, 135.66, 130.31, 130.13, 130.07, 128.63, 127.50, 117.83, 115.56, 107.27, 106.93, 104.03, 61.19, 60.95, 56.36, 56.27. HRMS (TOF-ESI) Calcd for C_30_H_27_N_5_O_6_, 553.1961 [M]^+^; observed: 375.2598 [M − C_10_H_12_O_3_]^+^.

The synthesis of compounds **5a**–**d** and **6a**–**d** and **11** was followed as per the procedure mentioned above, and their physical data were in agreement with reported values [25]. The data for unknown compounds are as follows:

#### 3.2.2. 1-Amino-4-(4-(dimethylamino)phenyl)imidazo[1,2-a]quinoxaline-2-carbonitrile (**5c**)

Yield: 72%, Color: red solid, m.p.: 169–171 °C, ^1^H-NMR (400 MHz, d_6_-DMSO) δ (ppm): 8.58–8.55 (3H, m), 7.89 (1H, dd, *J* = 8, 4 Hz), 7.55–7.51 (2H, m), 6.82–6.80 (4H, m), 2.99 (6H, s). Anal. calcd for C_19_H_16_N_6_: C, 69.50; H, 4.91; N, 25.59; Found: C, 69.41; H, 4.82; N, 25.40; MS (EI) 328 [M]^+^.

#### 3.2.3. 1-Amino-4-(4-isopropylphenyl)imidazo[1,2-a]quinoxaline-2-carbonitrile (**5d**)

Yield: 73%, Color: red solid, m.p.: 166–168 °C, ^1^H-NMR (400 MHz, d_6_-DMSO, TMS = 0) δ (ppm): 8.19 (1H, s), 7.90 (2H, d, *J* = 8 Hz), 7.83 (2H, s), 7.30 (2H, d, *J* = 8 Hz), 2.93–2.86 (1H, m), 1.18–1.17 (6H, d, *J* = 4 Hz). Anal. calcd for C_20_H_17_N_5_: C, 73.37; H, 5.23; N, 21.39; Found: C, 72.97; H, 5.01; N, 20.99.

#### 3.2.4. (*E*)-1-((3,4-Dimethoxybenzylidene)amino)-4-(3,4-dimethoxy-phenyl)imidazo[1,2-a]quinoxaline-2-carbonitrile (**6c**)

Yield: 72%, Color: red solid, m.p.: 170–172 °C, ^1^H-NMR (400 MHz, d_6_-DMSO, TMS = 0) δ (ppm): 9.04 (1H, s), 8.89–8.87 (1H, d, *J =* 8 Hz), 8.48–8.45 (1H, m), 8.22 (1H, s), 8.11–8.08 (1H, m), 7.73–7.72 (4H, m), 7.23–7.17 (2H, m), 3.90–3.85 (12H, m). HRMS (TOF-ESI) Calcd for C_28_H_23_N_5_O_4_, 493.1750 [M]^+^; observed: 494.1822 [M + H]^+^.

#### 3.2.5. Representative Procedure for the Synthesis of (*E*)-1-((4-Cyanobenzylidene)amino)-4-(4-cyanophenyl)-4,5-dihydroimidazo[1,2-a]quinoxaline-2-carbonitrile (**7k**)

To a suspension of **4** (0.240 g, 1.204 mmol) in MeOH was added 4-cyanobenzaldehyde (0.158 g, 1.204 mmol) and *p*-TsOH (1 mol %) as a catalyst. The mixture was heated in a sealed vial at 80 °C at 200W for 25 min under microwave irradiation (Biotage^®^ Initiator microwave synthesizer). Organic layer was to afford crude product and purified via column chromatography used to yield **7k**. Yield: 71%, Color: red solid, m.p.: 173–175 °C, ^1^H-NMR (400 MHz, *d_6_*-DMSO, TMS = 0) δ (ppm): 9.10 (1H, s), 8.18 (2H, d, *J* = 8 Hz), 8.04 (2H, d, *J* = 8 Hz), 7.85–7.81 (3H, m), 7.54 (2H, d, *J* = 8 Hz), 7.34 (1H, s), 7.12 (1H, t, *J* = 8 Hz), 7.01 (1H, d, *J* = 8.0 Hz), 6.80 (1H, t, *J* = 8 Hz), 5.91 (1H, s). HRMS (TOF-ESI) Calcd for C_26_H_15_N_7_, 425.1379 [M]^+^; observed: 426.1448 [M + H]^+^.

The synthesis of compounds **7a**–**k**, **8a**–**b**, **9a**–**c** and **10a**–**d** was followed as per the above mentioned procedure, and their physical data were in agreement with reported values [25]. The data for the unknown compound are as follows:

#### 3.2.6. 1-Amino-4-(2-nitrophenyl)-4,5-dihydroimidazo[1,2-*a*]quinoxaline-2-carbonitrile (**8c**)

Yield: 80%, Color: red solid, m.p.: 165–167 °C, ^1^H-NMR (400 MHz, d_6_-DMSO) δ (ppm): 8.02–8.00 (1H, m), 7.83 (1H, d, *J* = 8 Hz), 7.68 (1H, m), 7.42–7.45 (m, 1H), 7.52–7.51 (1H, m), 7.08–7.04 (1H, m), 6.99–6.96 (1H, m), 6.85–6.79 (2H, m), 6.30 (2H, s), 5.99 (1H, m). Anal. calcd for C_17_H_12_N_6_O_2_ C, 61.44; H, 3.64; N, 25.29; Found: C, 61.03; H, 3.54; N, 25.02.

### 3.3. Biology

#### 3.3.1. EGFR Inhibitory Assay

The test compounds were analyzed for EGFR inhibitory potential using z-lyte kinase assay kit–tyr 4 peptide assay kit (catalogue no. PV3193; Thermofisher, Mumbai, Maharashtra, India). The assay biochemically is FRET-based, which involves coupled enzyme format that relies on differential sensitivity of proteolytic cleavage of phosphorylated and non-phosphorylated peptides. The reaction proceeds in two steps, first involving the kinase reaction that is concerned with the transfer of phosphate group from ATP to single tyrosine residue, followed by development reaction, which involves site-protease role and cleaves non-phosphorylated peptide, allowing the disruption of FRET between donor and acceptor end of phosphorylated peptide, which facilitates deducing the emission ratio. The assay was performed per manufacturer protocol and per our published reports [16,31,32]. Briefly, the investigational compounds (including erlotinib as positive control) were tested at four varying concentrations of 100, 250, 500 and 720 nM and were added to assay plate in triplicate. Next, the assay master mix was prepared under ice-cold conditions by thawing and mixing kinase buffer (133 µL), kinase peptides (0.5 µL), phosphopeptide (0.5 µL) along with ATP (0.5 µL) solution. The master mix was added to testing compounds and further incubated for 1 h at room temperature and allowed the kinase reaction followed by development reaction to occur. After the stipulated period, reaction was stopped by addition of 5 µL stop solution to each reaction mixture. Furthermore, the emission ratio was determined spectrophotometrically (microplate reader) by measuring kinase inhibition at Ex/Em 400, 445 and 520 nm.

##### Calculations

Emission ratio: Emission ratio= Coumarin Emission (445 nm)/fluorescein emission (520 nm).

The extent of *phosphorylation* was calculated by the following formula (Equation (1)):

(1) $$\%\;phosphorylation\;=1-\;\frac{\left(Emission\;ratio\times \;F100\%\right)-\;C100\%}{\left(C0\%-C100\%\right)\;+\;\left[Emission\;ratio\;\left(F100\%-F0\%\right)\right]}$$

where *C*0% = Average coumarin emission signal of the 100% Phos. Control; *C*100% = Average coumarin emission signal of the 0% Phos. Control; *F*100% = Average Fluorescein emission signal of the 100% Phos. Control; *F*0% = Average Fluorescein emission signal of the 0% Phos. Control.

#### 3.3.2. Cell Culture and Maintenance

Cells such as A549, HCT-116 WT, MDA-MB-231 for the experiment were purchased from NCCS Pune, India and H1975 cell line was procured from American Type Cell Culture (ATCC, Manassas, VA, USA) and were deployed as per our published methodologies [17,31]. Briefly, cells were grown under sterile conditions using T-25 flasks or 100 mm dishes. The cells were supplemented with DMEM media, fetal bovine serum (FBS, 10%) and antibiotic solution (1X penicillin–streptomycin antibiotic solution) and incubated in a humidified atmosphere with a temperature of 37 °C enriched with 5% CO_2_ and 2% O_2_. Once the cells attained 80% confluency, they were either subcultured or utilized for experimental needs.

#### 3.3.3. 3-(4,5-Dimethythiazol-2-yl)-2,5-diphenyl Tetrazolium Bromide-Based MTT Assay

To assess the drug sensitivity against the cancer cells (A549, HCT-116 WT, MDA-MB-231 and H1975), cytotoxicity assay was performed using MTT reagent. Cells were seeded into a 96-well microplate at a density of 1 × 10⁴ cells per well and treated with various concentrations (0.5–20 μM) of gefitinib/erlotinib and/or test compounds. After a treatment period of 24–48 h, MTT reagent (3-[4, 5-dimethylthiazol-2-yl]-2, 5-diphenyltetrazolium bromide) was added to individual wells at a concentration of 0.5 mg/mL. The plates were wrapped in aluminum foil and incubated at 37 °C for 3 h. After this, the solution in each well was removed by suction. DMSO, as a solubilizing agent, was added to each well. The plates were then shaken and absorbance was measured at a dual-wavelength of 550 nm and 630 nm using a multimode automated microplate reader (Flex station III, Molecular Devices, Sunnyvale, CA, USA) and the results were expressed by percentage cell viability, assuming the viability of control cells as 100%. All measurements were performed in triplicate. The results were represented as mean ± SD, and dose–response curves were fitted using a nonlinear regression model with a sigmoidal dose–response and displayed graphically using GraphPad Prism version 5.00 for Windows (GraphPad Software, San Diego, CA, USA).

#### 3.3.4. Molecular Docking

Molecular docking studies were carried out using the Glide suite, Maestro 12.4 version of Schrodinger. All the designed molecules were drawn in a 2D format using ChemDraw Professional (Ver.15.0.0.106, PerkinElmer Inc., Waltham, MA, USA) and were imported in the Maestro to convert in 3D format using Ligprep. The force field used to prepare all the ligands was OPLS3 with ionization states at pH 7.0 ± 2.0, and tautomers were generated. The specified chirality was retained in the computation by generating at most 10 per ligand. The protein structures of the EGFR were imported in 3D form from the RCSB Protein Data Bank, PDB ID’s 3VJO (EGFR^WT^) and 5C8K (EGFR^L858R/T790M^). The proteins were prepared using Protein Preparation Wizard in which the protein was preprocessed; bond orders were assigned, explicit hydrogens were added, CCD database was used. In addition, disulphide bonds and zero-order bonds between metals and nearby atoms were created. After preprocessing, the workspace was analyzed and protonation was generated of the active chain selected. The protein was further refined by assigning optimization followed by restrained minimization. After preparing ligand and proteins, the grid was generated using receptor grid generation around an active site. Finally, molecular docking was performed by selecting prepared ligands and generated grid file. The flexible docking was carried out by selecting an extra precision mode, and RMSD was computed to input ligand geometries.

#### 3.3.5. Flow Cytometric-Based Analysis

BD C6 Accuri flow cytometer was employed to analyze critical anticancer assessment parameters, viz., alteration in ROS, mitochondrial membrane permeability (∆ψm), mode of cell death and progression of cell cycle upon treatment of **6b** at the sub-IC_50_ concentration for 48 h to A549 cancer cells. The assays were performed following our previous published reports [16,32,33]. Briefly, A549 cells were cultured in 100 mm dishes and maintained to attain at least 80% confluency. Once attained, dishes were treated with **6b** and were incubated for 48 h. After the stipulated period, the remaining media were collected along with the cells (trypsinization) and were centrifuged (1200 rpm for 5 min) and washed two times using 1X PBS. Furthermore, the cells were raised to the concentration of 400 µL and were equally divided (5 × 10^4^) in four aliquots. The first three aliquots were added with H_2_DCFDA (10 µM/100 µL), JC-1 dye (1 mM/mL) and Annexin V and PI (as per manufacturer protocol (Thermo Fisher: V13242, Maharashtra, India) to analyze detection of alteration in ROS, ∆ψm and mode of cell death, respectively. The cancer cells were incubated in the dark for 30 min at rt before analysis. Next, the fourth aliquot cells were fixed using chilled ethanol (70%) and incubated at −20 °C for 4 h to allow fixing. After 4 h, cells were centrifuged (2200 rpm for 10 min) and washed twice with 1X PBS. Furthermore, the cells were treated with RNase and PI was added and they were further incubated for 30 min in the dark before analysis. All the samples were analyzed using BD C6 flow cytometer using various FL channels.

### 3.4. Statistical Treatment

All the data were represented as mean ± SD or mean ± SEM of three independent experiments. One-way ANOVA was used for the comparison of multiple groups (Table 1). Statistical difference was set at *p* < 0.05 between various treated groups. Two-way ANOVA was used for the comparison of multiple groups for data of Figure 4B. For statistical analysis of the data (Figure 4), GraphPad Prism 8.0.2 software (San Diego, CA, USA) was used.

## 4. Conclusions

In summary, we rationally designed and synthesized 30imidazoquinoxaline-based non-covalent EGFR inhibitors. Among all the screened compounds, five compounds exhibited comparable anti-EGFR^WT^ activity regarding positive control and potent antiproliferative activity against cell lines including lung, colon and breast harboring EGFR^WT^. Three compounds, **6b**, **7j** and **9a**, presented excellent inhibitory activity against gefitinib-resistant NSCLC cell line H1975 harboring EGFR^L858R/T790M^, indicating their potential against EGFR mutant cancer. Molecular docking studies rationalized the basis of EGFR inhibitory activity as compounds were able to interact with both Thr790 (wild type) and Met790 (mutant type) gatekeeper residues and with conserved catalytic Lys745 residue at a shorter distance (mutant type).

Furthermore, **6b** emerged as the most promising anti-EGFR inhibitor with a great effect on gefitinib-resistant H1975 lung cancer cells, decreased oxidative stress and altered mitochondrial membrane potential leading to apoptosis and induced cell cycle arrest in A549 lung cancer cells at G phase. In vivo studies using **6b** are underway, and the results will be published in due course.

## Figures and Tables

**Table 1:** In vitro EGFR inhibitory activity of compounds **5**–**11**.

Compound	EGFR Inhibition IC_50_ (nM) ± SEM ^a,b^	Compound	EGFR Inhibition IC_50_ (nM) ^a^ ± SEM ^a,b^
**5a**	>720	**7i**	613.37 ± 0.024
**5b**	244.45 ± 0.032	**7j**	193.18 ± 0.021
**5c**	>720	**7k**	>720
**5d**	>720	**8a**	>720
**6a**	429.81 ± 0.018	**8b**	>720
**6b**	211.22 ± 0.027	**8c**	>720
**6c**	>720	**9a**	223.32 ± 0.031
**6d**	>720	**9b**	244.51 ± 0.021
**7a**	>720	**9c**	221.53 ± 0.028
**7b**	>720	**10a**	247.32 ± 0.021
**7c**	620.17 ± 0.019	**10b**	>720
**7d**	652.56 ± 0.024	**10c**	716.32 ± 0.026
**7e**	665.89 ± 0.029	**10d**	624.57 ± 0.024
**7f**	232.40 ± 0.031	**11**	669.39 ± 0.019
**7g**	476.87 ± 0.022	Erlotinib	221.03 ± 0.028
**7h**	222.21 ± 0.028		

^a^ Assay was performed in triplicate. ^b^ Data are presented as means ± standard error mean (S.E.M.) of three independent experiments. One-way ANOVA was used for comparison of multiple groups. Statistical difference was set at *p* < 0.05 between various treated groups.

**Table 2:** Antiproliferative potential of **6b**, **7h**, **7j**, **9a** and **9c** against human cancer cells.

Code	A549 (Lung Cancer)	HCT-116 WT (Colon Cancer)	MDA-MB-231 (Breast Cancer)
	IC_50_ (μM) ± SEM ^a,b^		
**6b**	2.7 ± 0.032	5.1 ± 0.029	4.1 ± 0.031
**7h**	4.09 ± 0.024	<1	11.2 ± 0.022
**7j**	8.75 ± 0.028	<1	2.2 ± 0.026
**9a**	6.63 ± 0.031	>25	14.1 ± 0.021
**9c**	12.06 ± 0.021	7.90 ± 0.027	13.6 ± 0.028
Erlotinib	4.56 ± 0.019	2.98 ± 0.023	3.33 ± 0.018

^a^ Assay was performed in triplicate, and data were compiled for 48 h incubation. ^b^ Data are presented as means ± standard error mean (S.E.M.) of three independent experiments.

## Data Availability

The data presented in this study are available on request from the corresponding author.

## Supplementary Materials

The following are available online: Figure S1: 3D docking pose of A. **I** and B. **II**, at EGFR ATP kinase domain; Figure S2: Bar graphs representing percentage survival of normal cells, **A.** HBL-100 (breast); **B.** HPBMCs (Human Peripheral Blood Mononuclear Cells), upon treatment with investigational compounds **6b**, **7h**, **7j**, **9a** and **9c** at 10 µM concentration previously incubated for 24 h; Figure S3: Bar graphs representing alteration in ROS as indicated by H2DCFDA assay in **A.** MCF-7; **B.** MDA-MB-231 cells. The assays were performed in cancer cells previously treated with investigational compound **6b** at sub-IC_50_ concentration for 48 h before analysis; Figures S4–S11: Spectra of the representative compounds; Table S1: Docking scores of **I**, **II**, erlotinib and designed compound **6b**.

## Sample Availability

Samples of the compounds are not available from the authors.

## References

[1] Wee P., Wang Z. (2017). Epidermal growth factor receptor cell proliferation signaling pathways. Cancers.

[2] Kumar M., Joshi G., Chatterjee J., Kumar R. (2020). Epidermal Growth Factor Receptor and its Trafficking Regulation by Acetylation: Implication in Resistance and Exploring the Newer Therapeutic Avenues in Cancer. Curr. Top. Med. Chem..

[3] Shepherd F.A., Rodrigues Pereira J., Ciuleanu T., Tan E.H., Hirsh V., Thongprasert S., Campos D., Maoleekoonpiroj S., Smylie M., Martins R. (2005). Erlotinib in previously treated non–small-cell lung cancer. N. Engl. J. Med..

[4] Paez J.G., Jänne P.A., Lee J.C., Tracy S., Greulich H., Gabriel S., Herman P., Kaye F.J., Lindeman N., Boggon T.J. (2004). EGFR mutations in lung cancer: Correlation with clinical response to gefitinib therapy. Science.

[5] Cheng H., Nair S.K., Murray B.W. (2016). Recent progress on third generation covalent EGFR inhibitors. Bioorg. Med. Chem. Lett..

[6] Grabe T., Lategahn J., Rauh D. (2018). C797S Resistance: The undruggable EGFR mutation in non-small cell lung cancer?. ACS Med. Chem. Lett..

[7] Lacouture M.E. (2006). Mechanisms of cutaneous toxicities to EGFR inhibitors. Nat. Rev. Cancer.

[8] Gray N., Jaebong J., De Clercq D., Eck M., Janne P. (2017). Bifunctional Molecules for Degradation of EGFR and Methods of Use.

[9] Niederst M.J., Hu H., Mulvey H.E., Lockerman E.L., Garcia A.R., Piotrowska Z., Sequist L.V., Engelman J.A. (2015). The allelic context of the C797S mutation acquired upon treatment with third-generation EGFR inhibitors impacts sensitivity to subsequent treatment strategies. Clin. Cancer Res..

[10] Li Q., Zhang T., Li S., Tong L., Li J., Su Z., Feng F., Sun D., Tong Y., Wang X. (2019). Discovery of potent and non-covalent reversible EGFR kinase inhibitors of EGFRL858R/T790M/C797S. ACS Med. Chem. Lett..

[11] Kalra S., Joshi G., Kumar M., Arora S., Kaur H., Singh S., Munshi A., Kumar R. (2020). Anticancer potential of some imidazole and fused imidazole derivatives: Exploring the mechanism via epidermal growth factor receptor (EGFR) inhibition. RSC Med. Chem..

[12] Garg M., Chauhan M., Singh P.K., Alex J.M., Kumar R. (2015). Pyrazoloquinazolines: Synthetic strategies and bioactivities. Eur. J. Med. Chem..

[13] Kaur G., Cholia R.P., Joshi G., Amrutkar S.M., Kalra S., Mantha A.K., Banerjee U.C., Kumar R. (2018). Anti-cancer activity of dihydropyrazolo [1, 5-c] quinazolines against rat C6 glioma cells via inhibition of topoisomerase II. Arch. Pharm..

[14] Kumar D., Kaur G., Negi A., Kumar S., Singh S., Kumar R. (2014). Synthesis and xanthine oxidase inhibitory activity of 5, 6-dihydropyrazolo/pyrazolo [1, 5-c] quinazoline derivatives. Bioorg. Chem..

[15] Kumar D., Kumar R. (2014). Microwave-assisted synthesis of pyrazolo [1, 5-c] quinazolines and their derivatives. Tetrahedron Lett..

[16] Sawant D.M., Sharma S., Pathare R.S., Joshi G., Kalra S., Sukanya S., Maurya A.K., Metre R.K., Agnihotri V.K., Khan S. (2018). Relay tricyclic Pd (ii)/Ag (i) catalysis: Design of a four-component reaction driven by nitrene-transfer on isocyanide yields inhibitors of EGFR. Chem. Commun..

[17] Ansari A.J., Joshi G., Yadav U.P., Maurya A.K., Agnihotri V.K., Kalra S., Kumar R., Singh S., Sawant D.M. (2019). Exploration of Pd-catalysed four-component tandem reaction for one-pot assembly of pyrazolo [1, 5-c] quinazolines as potential EGFR inhibitors. Bioorg. Chem..

[18] Negi A., Alex J.M., Amrutkar S.M., Baviskar A.T., Joshi G., Singh S., Banerjee U.C., Kumar R. (2015). Imine/amide–imidazole conjugates derived from 5-amino-4-cyano-N1-substituted benzyl imidazole: Microwave-assisted synthesis and anti-cancer activity via selective topoisomerase-II-α inhibition. Bioorg. Med. Chem..

[19] El Newahie A.M., Ismail N.S., Abou El Ella D.A., Abouzid K.A. (2016). Quinoxaline-Based Scaffolds Targeting Tyrosine Kinases and Their Potential Anti-cancer Activity. Arch. Pharm..

[20] Chouchou A., Patinote C., Cuq P., Bonnet P.-A., Deleuze-Masquéfa C. (2018). Imidazo [1, 2-a] quinoxalines derivatives grafted with amino acids: Synthesis and evaluation on A375 melanoma cells. Molecules.

[21] Moarbess G., Deleuze-Masquefa C., Bonnard V., Gayraud-Paniagua S., Vidal J.-R., Bressolle F., Pinguet F., Bonnet P.-A. (2008). In vitro and in vivo anti-tumoral activities of imidazo [1, 2-a] quinoxaline, imidazo [1, 5-a] quinoxaline, and pyrazolo [1, 5-a] quinoxaline derivatives. Bioorg. Med. Chem..

[22] Moarbess G., Guichou J.-F., Paniagua-Gayraud S., Chouchou A., Marcadet O., Leroy F., Ruédas R., Cuq P., Deleuze-Masquéfa C., Bonnet P.-A. (2016). New IKK inhibitors: Synthesis of new imidazo [1, 2-a] quinoxaline derivatives using microwave assistance and biological evaluation as IKK inhibitors. Eur. J. Med. Chem..

[23] Chen P., Iwanowicz E.J., Norris D., Gu H.H., Lin J., Moquin R.V., Das J., Wityak J., Spergel S.H., de Fex H. (2002). Synthesis and SAR of novel imidazoquinoxaline-based Lck inhibitors: Improvement of cell potency. Bioorg. Med. Chem. Lett..

[24] Sharma S., Joshi G., Kalra S., Singh S., Kumar R. (2018). Synthetic versus enzymatic pictet-spengler reaction: An overview. Curr. Org. Synth..

[25] Joshi G., Chauhan M., Kumar R., Thakur A., Sharma S., Singh R., Wani A.A., Sharon A., Bharatam P.V., Kumar R. (2018). Cyclocondensation reactions of an electron deactivated 2-aminophenyl tethered imidazole with mono/1, 2-biselectrophiles: Synthesis and DFT studies on the rationalization of imidazo [1, 2-a] quinoxaline versus benzo [f] imidazo [1, 5-a][1, 3, 5] triazepine selectivity switches. Org. Chem. Front..

[26] Kumar R., Ujjinamatada R.K., Hosmane R.S. (2008). The first synthesis of a novel 5: 7: 5-fused diimidazodiazepine ring system and some of its chemical properties. Org. Lett..

[27] Bissantz C., Kuhn B., Stahl M. (2010). A medicinal chemist’s guide to molecular interactions. J. Med. Chem..

[28] Heald R., Bowman K.K., Bryan M.C., Burdick D., Chan B., Chan E., Chen Y., Clausen S., Dominguez-Fernandez B., Eigenbrot C. (2015). Non-covalent mutant selective epidermal growth factor receptor inhibitors: A lead optimization case study. J. Med. Chem..

[29] Orphanos G.S., Ioannidis G.N., Ardavanis A.G. (2009). Cardiotoxicity induced by tyrosine kinase inhibitors. Acta Oncol..

[30] Force T., Kolaja K.L. (2011). Cardiotoxicity of kinase inhibitors: The prediction and translation of preclinical models to clinical outcomes. Nat. Rev. Drug Discov..

[31] Joshi G., Kalra S., Yadav U.P., Sharma P., Singh P.K., Amrutkar S., Ansari A.J., Kumar S., Sharon A., Sharma S. (2020). E-pharmacophore guided discovery of pyrazolo [1, 5-c] quinazolines as dual inhibitors of topoisomerase-I and histone deacetylase. Bioorg. Chem..

[32] Chauhan M., Joshi G., Kler H., Kashyap A., Amrutkar S.M., Sharma P., Bhilare K.D., Banerjee U.C., Singh S., Kumar R. (2016). Dual inhibitors of epidermal growth factor receptor and topoisomerase IIα derived from a quinoline scaffold. RSC Adv..

[33] Joshi G., Nayyar H., Kalra S., Sharma P., Munshi A., Singh S., Kumar R. (2017). Pyrimidine containing epidermal growth factor receptor kinase inhibitors: Synthesis and biological evaluation. Chem. Biol. Drug Des..

