# Supplementary material for: Design and Synthesis of Non-Covalent Imidazo[1,2-a]quinoxaline-Based Inhibitors of EGFR and Their Anti-Cancer Assessment
Source: Molecules. 2021 Mar 9;26(5):1490. doi: 10.3390/molecules26051490 (PMC7967119; doi:10.3390/molecules26051490)

molecules

MDPI

Supporting Information

Design and Synthesis of Non-Covalent Imidazo[1,2-*a*]quinoxaline-Based Inhibitors of EGFR and Their Anti-Cancer Assessment

Manvendra Kumar [1], Gaurav Joshi [1,2], Sahil Arora [1], Tashvinder Singh [3], Sajal Biswas [1], Nisha Sharma [4], Zahid Rafiq Bhat [4], Kulbhushan Tikoo [4], Sandeep Singh [3,*] and Raj Kumar [1,*]

[1] Laboratory for Drug Design and Synthesis, Department of Pharmaceutical Sciences and Natural Products, School of Health Sciences, Central University of Punjab, Bathinda 151401, India; pharma.manvendra003@gmail.com (M.K.); garvjoshi@gehu.ac.in (G.J.); deepsahil7999@gmail.com (S.A.); sajal.143rx@gmail.com (S.B.)
[2] School of Pharmacy, Graphic Era Hill University, Dehradun 248171, India
[3] Department of Human Genetics and Molecular Medicine, Central University of Punjab, Bathinda 151401 Punjab, India; sidhu.tash@gmail.com (T.S.); sandeepsingh82@gmail.com (S.S.)
[4] Department of Pharmacology and Toxicology, National Institute of Pharmaceutical Education and Research, S.A.S. Nagar, Punjab 160062, India; nishasharma.nics8@gmail.com (N.S.); zbhat800@gmail.com (Z.R.B.); tikoo.k@gmail.com (K.T.)
* Correspondence: raj.khunger@gmail.com or raj.khunger@cup.edu.in

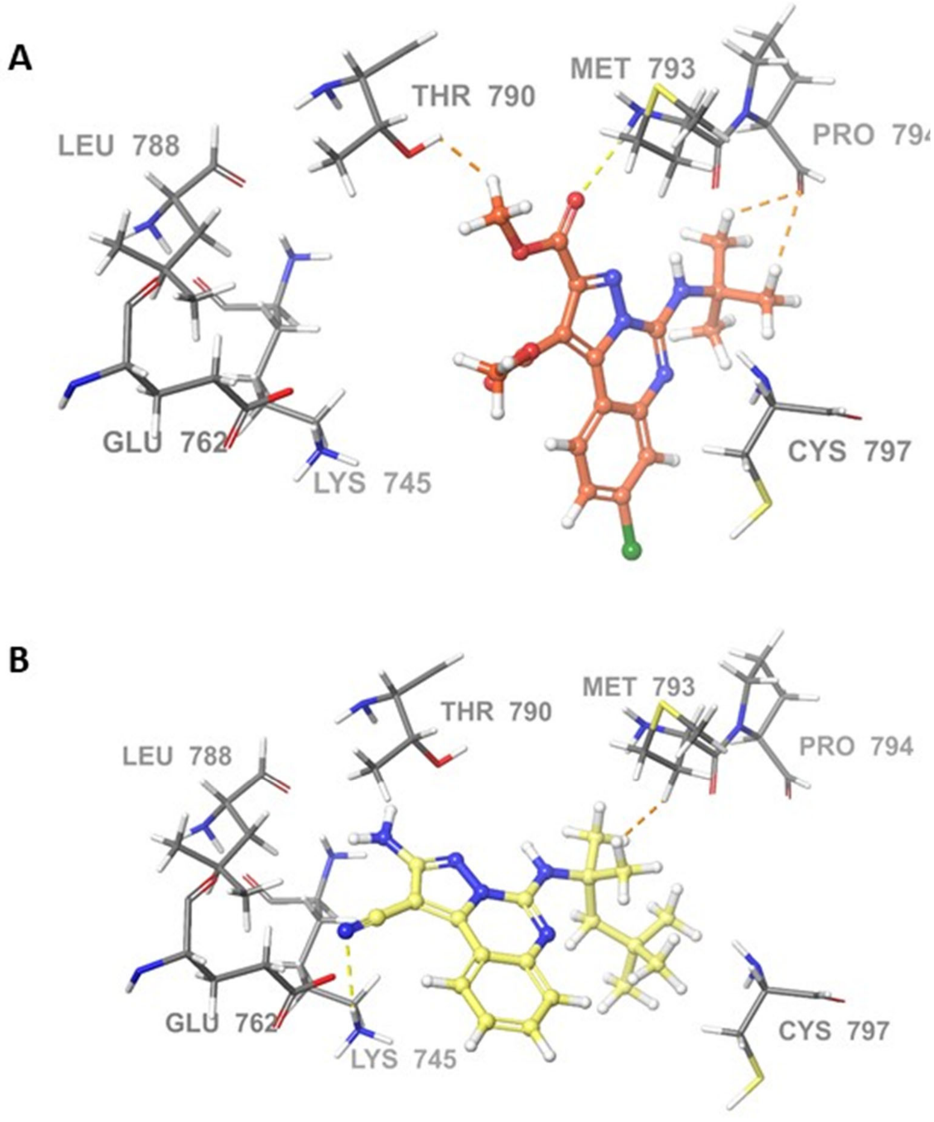

Figure S1. 3D docking pose of **A. I** and **B. II**, at EGFR ATP kinase domain.

Table S1. Docking scores of **I**, **II**, erlotinib and designed compound **6b.**

Entry	Compound	Dock score
1	6b	−5.463
2	**I**	−4.950
3	**II**	−4.067
4	Erlotinib	−4.985

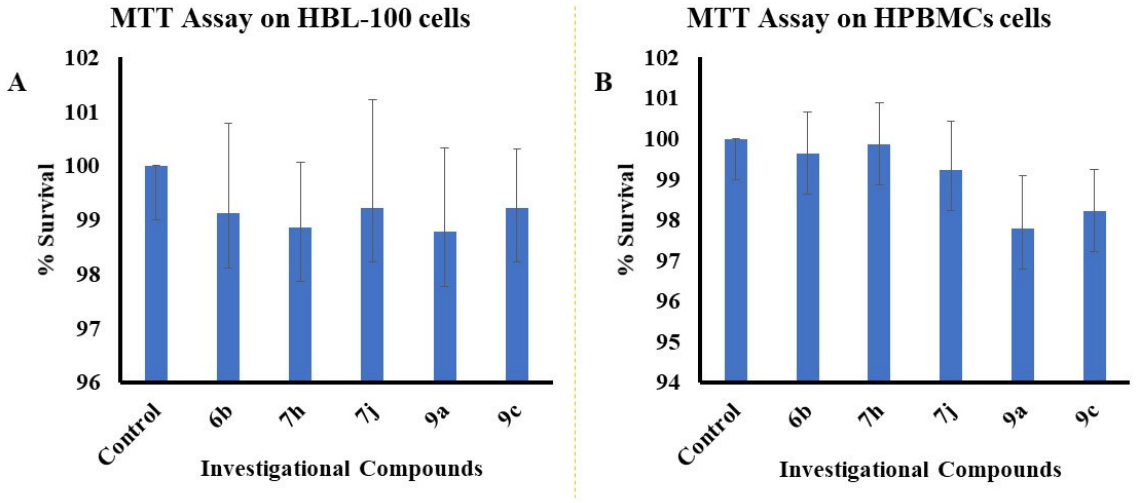

Figure S2. Bar graphs representing percentage survival of normal cells, **A.** HBL-100 (breast); **B.** HPBMCs (Human Peripheral Blood Mononuclear Cells), upon treatment with investigational compounds **6b, 7h, 7j, 9a** and **9c** at 10 μM concentration previously incubated for 24 h.

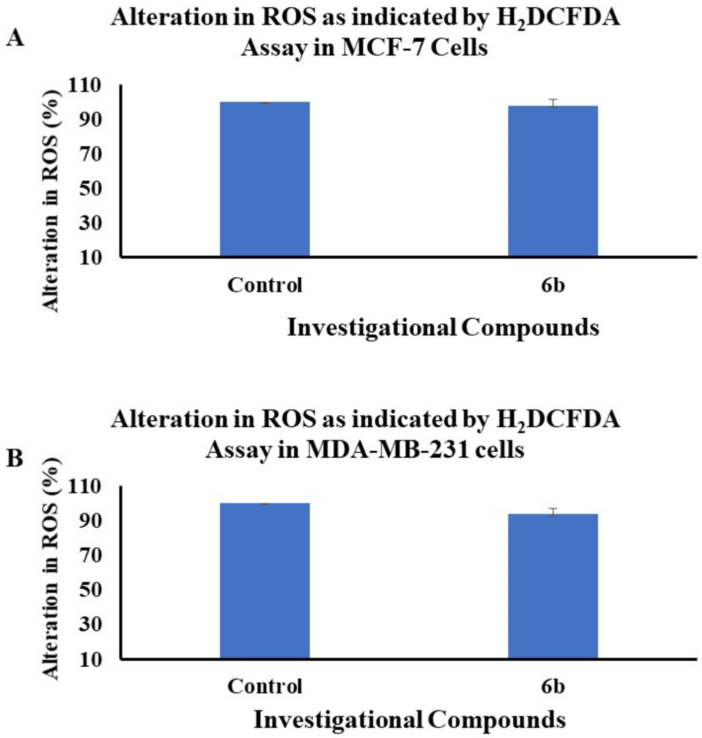

Figure S3. Bar graphs representing alteration in ROS as indicated by H_2DCFDA assay in **A.** MCF-7; **B.** MDA-MB-231 cells. The assays were performed in cancer cells previously treated with investigational compound **6b** at sub-IC_{50} concentration for 48 h before analysis.

Spectra for representative compounds (Figures S4–S11)

^{1}H-NMR

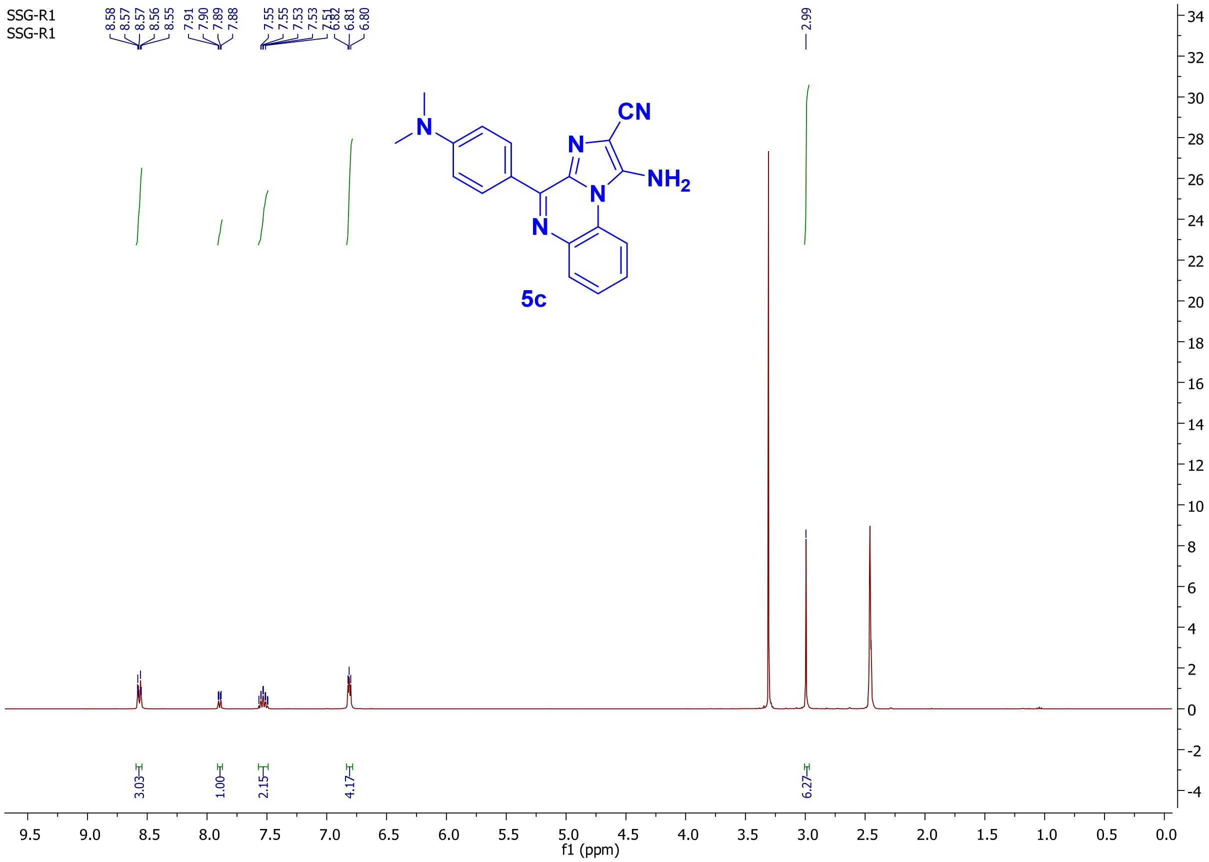

Mass Spectrometry

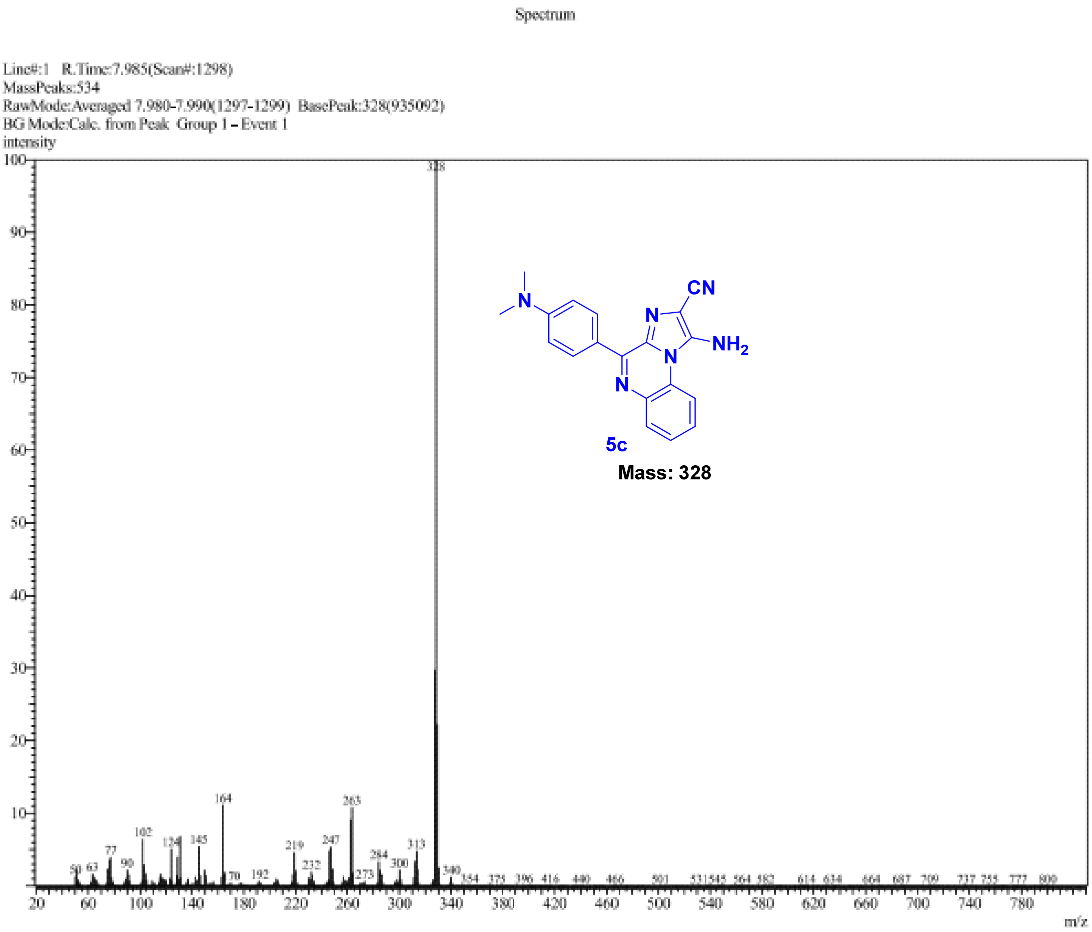

¹H-NMR

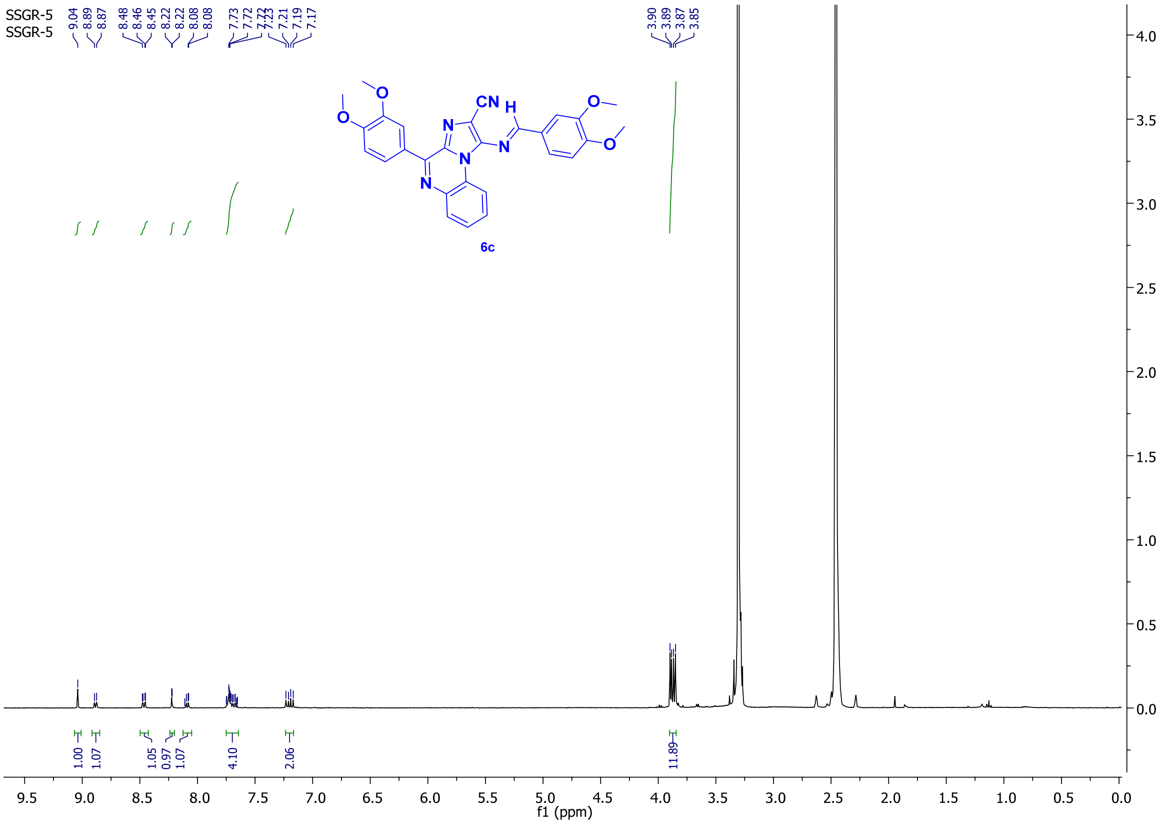
SSGR-5
SSGR-5
9.04
8.89
8.87
8.48
8.46
8.45
8.22
8.22
8.08
8.08
7.73
7.72
7.22
7.23
7.21
7.19
7.17
3.90
3.89
3.87
3.85
CN
H
O
N
6c
1.00
1.07
1.05
0.97
1.07
4.10
2.06
11.89
f1 (ppm)

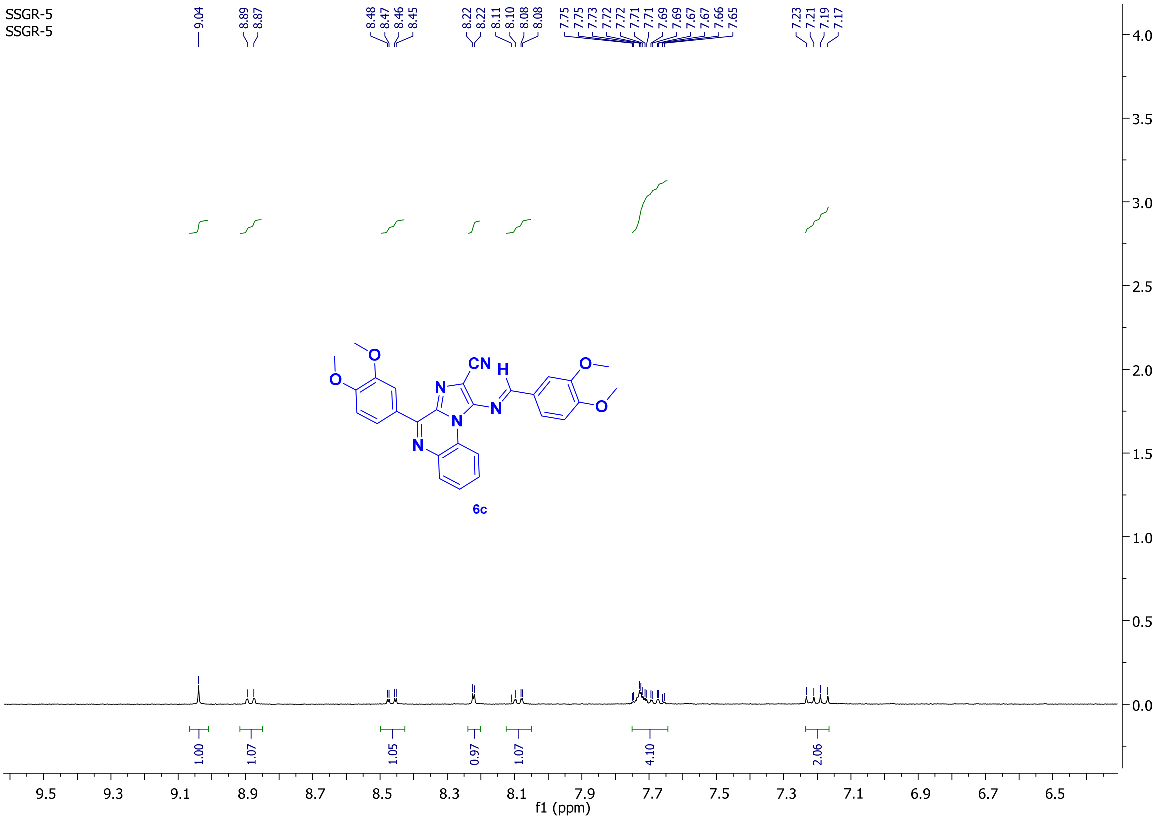

Mass Spectrometry

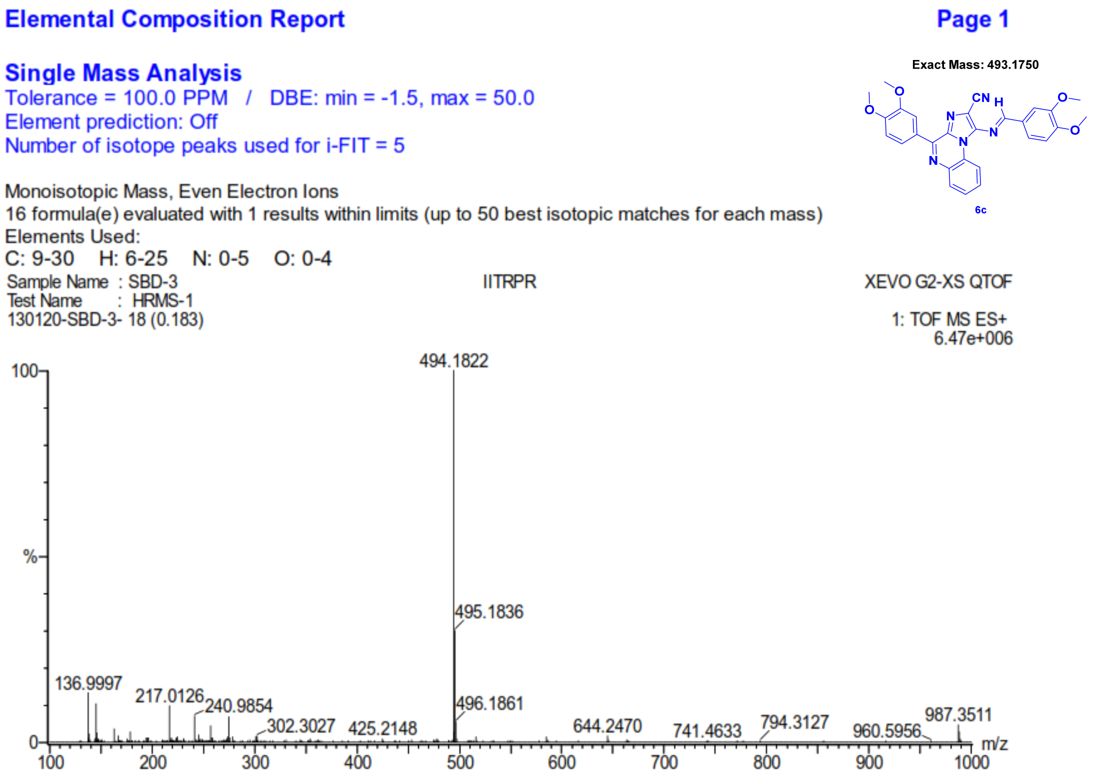

^{1}H-NMR

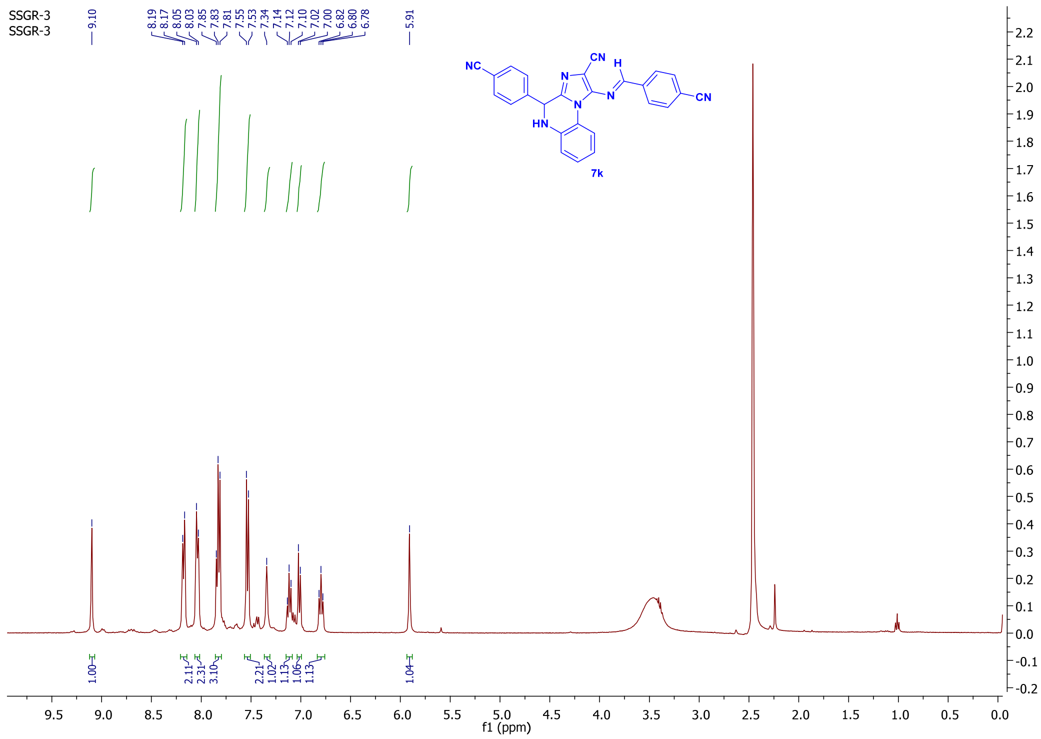

Mass Spectrometry

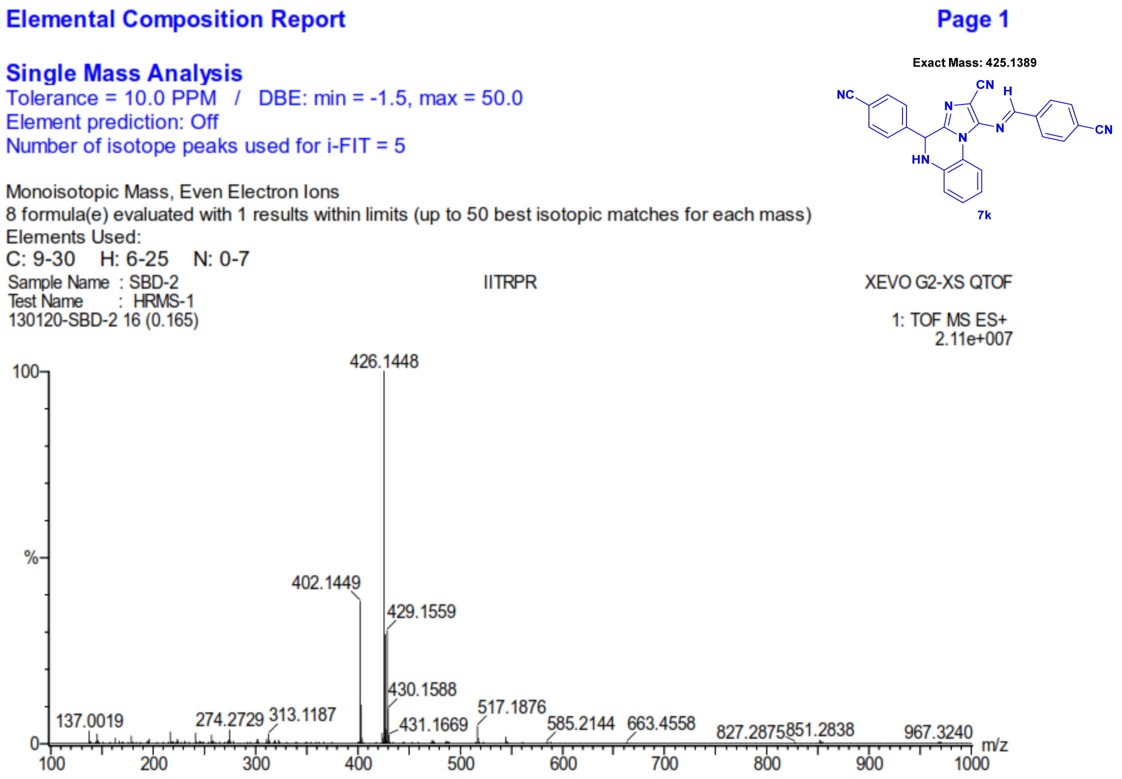

^{1}*H-NMR*

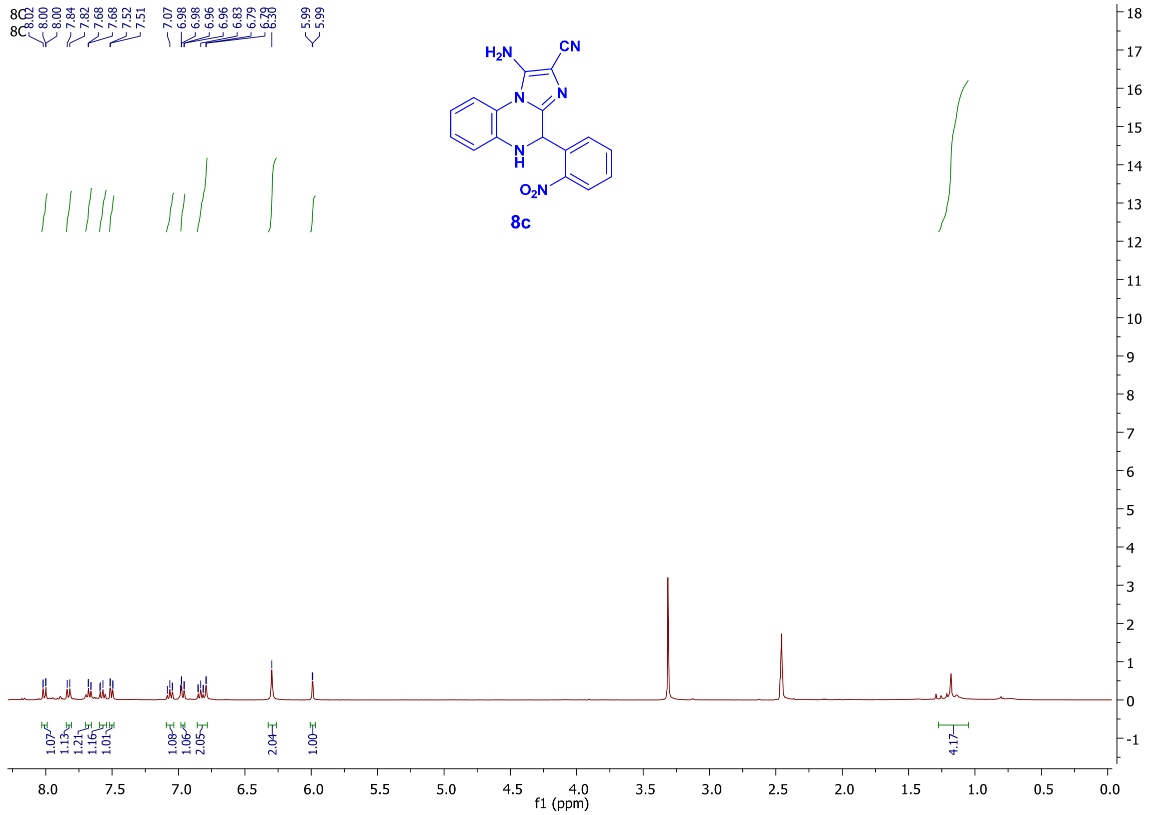

Supplement: Supplementary file 1 [file molecules-26-01490-s001.pdf]
